# Estimating the predictive value of negative severe acute respiratory coronavirus virus 2 (SARS-CoV-2) results: A prospective study

**DOI:** 10.1017/ice.2020.1362

**Published:** 2020-12-10

**Authors:** David Hirschwerk, Mathew Foley, Martin Lesser, Bruce Farber, James M. Crawford, Karina W. Davidson, Gregory J. Berry, Elizabeth Smith, Charles Kast, Vladimir Volel, Thomas McGinn

**Affiliations:** 1 Division of Infectious Diseases, Department of Medicine, North Shore University Hospital, Manhasset and Long Island Jewish Medical Center, New Hyde Park, New York; 2 Donald and Barbara Zucker School of Medicine at Hofstra/Northwell, Hempstead, New York; 3 Department of Emergency Medicine, North Shore University Hospital, Manhasset, New York; 4 Institute of Health Innovations and Outcomes Research, Feinstein Institutes for Medical Research, Northwell Health, Manhasset, New York; 5 Infectious Disease Diagnostics, Northwell Health Laboratories, Little Neck, New York; 6 Department of Medicine, North Shore University Hospital, Manhasset, New York

## Abstract

We performed a prospective study of 501 patients, regardless of symptoms, admitted to the hospital, to estimate the predictive value of a negative nasopharyngeal swab for severe acute respiratory coronavirus virus 2 (SARS-CoV-2). At a positivity rate of 10.2%, the estimated negative predictive value (NPV) was 97.2% and the NPV rose as prevalence decreased during the study.

Establishing a diagnosis of coronavirus disease 2019 (COVID-19) is based on reverse-transcriptase polymerase chain reaction (RT-PCR) testing for severe acute respiratory coronavirus virus 2 (SARS-CoV-2) from a sample obtained using a nasopharyngeal swab (NP swab).^1^ False-negative results occur in 27%–37% of these samples.^2,3^

False-negative results occur when the laboratory platform does not detect SARS-CoV-2 RNA in samples confirmed to contain the virus.^2,3,5^ But a false-negative result also occurs when insufficient viral RNA is present on the NP swab.^5,6^ This insufficiency is related to the operator performance of the swab and the cooperation of the patient undergoing testing.

The likelihood that a negative NP swab reflects the absence of SARS-CoV-2 infection (ie, negative predictive value, NPV) is related to illness prevalence in the community. The NPV has not been prospectively derived to date, and having a more complete understanding of this dynamic is critical for clinical decision making as testing strategies evolve during the pandemic.

The primary objective of this study was to estimate the NPV for an NP swab. A secondary aim was to estimate the change in NPV as prevalence changed. Our protocol was based upon prospectively performing 2 NP swabs on patients admitted to the hospital through the emergency department and randomly assigning one swab as the index sample.

## Methods

The study was approved by the institutional review boards of the Northwell Health System and the Feinstein Institute with a waiver of informed consent. The study was performed at North Shore University Hospital in Manhasset, New York, a 760-bed tertiary-care hospital that is part of the Northwell Health System.

From April 20 onward, all patients admitted to the hospital from the emergency department, regardless of symptoms, underwent a single NP swab for SARS-CoV-2 testing. Data collection for this study occurred from May 6 through June 18, 2020. In total, 501 patients were included. Patients were included consecutively over several time points during the period, but breaks in inclusion occurred because of concerns about supply (ie, swabs and viral media) and laboratory capacity. The NP swabbing procedures were performed by either emergency department physicians, advanced care providers, or nurses.

Two NP swabs were collected at the same sitting from patients included in this study, and the operator chose which nostril to swab. After the swabbing procedure, each swab was placed in a separate tube of viral media and was sent for testing at the central Northwell Health Laboratories.^7^ All 1,002 samples in this study were analyzed on the same platform, which had been previously validated and reported using clinical samples (Hologic Panther Fusion, Marlborough, MA).^8^

Using a random number generator, 1 of the 2 samples from each patient was designated as the index swab. If the result of the index swab was positive, the patient was considered positive for SARS-CoV-2. If the index swab was negative, then the result of the second NP swab was revealed. If the second NP swab was positive, the negative result on the index NP swab was declared a false negative.

A patient was considered to have COVID-19 if at least 1 of the 2 NP swabs was positive (true positives plus false negatives). We calculated the positivity rate as a percentage, which is considered a reflection of prevalence.

The NPV was computed as follows: 1 − (the number of false negative results divided by the number of negative index swab results). The exact binomial 95% confidence intervals were also computed.

To account for changing NPV rates over time, this 44-day interval was divided into equal consecutive periods of 11 days each. No dual swabs were collected during period 3 because availability of testing materials was reduced at our hospital during this period and only 1 swab per patient could be allocated. The calculated positivity rate for each period was compared to the actual system-wide Northwell Health System positivity rate among patients evaluated in the emergency department and the hospital for the same period.

## Results

For the May 6–June 18 study period, 501 study patients were tested using 1,002 swabs. Of the 501 study patients, 51 (10.2%) had a positive result on either of the 2 NP swabs. Moreover, 38 index swabs were positive (7.6%; 95% CI, 5.4%–10.3%). Of the 463 negative index NP swabs, 13 had a corresponding positive result on the second NP swab (ie, a false-negative result), yielding an index swab NPV of [1 − (13/463)] or 97.2% (95% CI, 95.69%–98.50%) (Table 1).

**Table 1.** Viral Detection, Negative Predictive Value (NPV) and Positivity Rate for Index and Second Swabs by Time Interval and Total

Index Swab Result	Second Swab Result	Period 1, No.	Period 2, No.	Period 3, No.	Period 4, No.	Total, No.
Neg	Neg	202	120	N/A	128	450
Neg	Pos	8	4	N/A	1	13
Pos	Neg	11	5	N/A	2	18
Pos	Pos	14	3	N/A	3	20
Estimated NPV, %		96.2	96.8	N/A	99.2	97.2
Estimated % positivity		14.0	9.1	N/A	4.5	10.2

Note. Neg, negative; pos, positive.

**Fig. 1.**
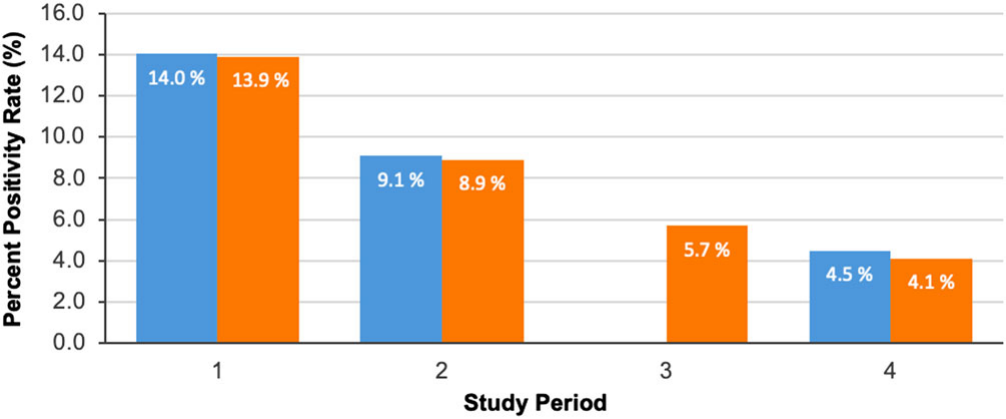
Positivity rates of the study population and the overall health system. 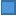, Calculated % Positivity Rate in Study Population; 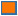, % Positivity Rate in Northwell Health System (Emergency Dept. and Hospitalized Patients).

Over the 4 periods, the estimated NPVs were 96.2% (95% CI, 92.6%–98.30%) for period 1, 96.8% (95% CI, 92.0%–99.10%) for period 2, not available for period 3, and 99.2% (95% CI, 95.8%–99.9%) for period 4. These findings indicate an upward trend in NPV, as would be expected by the decreasing prevalence of COVID-19 across the study period. The corresponding positivity rates were 14.0% for period 1, 9.1% for period 2, not applicable for period 3, and 4.5% for period 4. The SARS-CoV-2 positivity rate for the health system emergency department and hospitalized patients during these 4 intervals was 13.9% (1,473 positives of 10,597 patients) for period 1, 8.9% (1,056 of 11,872 patients) for period 2, 5.7% (726 of 12,769 patients) for period 3, and 4.1% (519 of 12,768 patients) for period 4 (Fig. 1). The calculated positivity rate for the three 11-day intervals during which 2 swabs could be performed in this study, correlates significantly with the actual positive test rate for our health system (R^2^ > 0.99; *P* < .01).

## Discussion

A major strength of our study was the method of prospectively performing dual NP swabs on all patients regardless of symptoms. We demonstrated that false-negative test results occur and that the estimated NPV correlates with prevalence.

In our study, in which both NP swabs were performed in the same sitting, several factors that should have led to both swabs yielding the same outcome: (1) The quantity of virus in the nasopharynx would be expected to be the same, (2) specimens were processed in the same manner, and (3) the testing platform for each specimen was the same. Therefore, we believe that a major influence for false-negative results for the index samples is the quality of the sample, which is affected by the technique of the operator and the cooperation of the patient.

This study has several limitations. It was initiated after the peak of SARS-CoV-2 test prevalence positivity rates in the New York region. During the entire period that our study considered, just >10% of patients had a positive result, and prevalence during the overall study period. However, our findings remain applicable because many population prevalence rates being encountered now in the United States are 10% or lower.^9^

Although predictive value is influenced by illness prevalence, it is also affected by clinical signs and symptoms. These data were not included in this study. However, many centers, like ours, are currently screening all patients admitted to their hospitals. Additionally, as testing evolves and expands in the community, most patients tested will not have symptoms of COVID-19, but will be tested as part of broader screening strategies. At the prevalence rates observed in our study, we believe that our findings can be applied.

In summary, we performed a study of 501 patients to prospectively estimate the predictive value of negative NP swabs during periods of changing prevalence of infection. The overall NPV of >97% occurred during an period in which the estimated positivity rate was 10%, and the study cohort positivity rates matched the overall positivity rates encountered by our health system during the same periods.

False-negative results and NPV have yet to be estimated by a prospective design like we have performed. We believe that comprehensively understanding test attributes is critical as the pandemic evolves. Our study design demonstrates that sample quality influences the occurrence of false-negative results and that opportunities exist to optimize sampling quality.
